# How well can we predict educational outcomes? Examining the roles of cognitive ability and social position in educational attainment

**DOI:** 10.1080/21582041.2016.1138502

**Published:** 2016-02-23

**Authors:** Tim Morris, Danny Dorling, George Davey Smith

**Affiliations:** ^a^ School of Geographical Sciences, University of Bristol, Bristol, UK; ^b^ School of Geography and the Environment, University of Oxford, Oxford, UK; ^c^ MRC Integrative Epidemiology Unit (IEU) at the University of Bristol, School of Social and Community Medicine, Bristol, UK

**Keywords:** social inequality, education, schools, cognitive ability, ALSPAC

## Abstract

Social inequalities in UK educational outcomes continue to persist despite improvements in recent years. However, studies that examine these inequalities fail to account for differences in prior cognitive ability. We seek to determine the influence of cognitive ability on educational outcomes and the extent of socio-economic disparities in education across a wide range of indicators while accounting for cognitive ability. Social inequalities exist whereby children from disadvantaged backgrounds systematically underperform compared to their advantaged peers regardless of cognitive ability; high ability children from disadvantaged backgrounds are disproportionately less likely to attain good grades compared to children from advantaged backgrounds. In addition, school effects operate to add to this inequality as children in fee-paying secondary schools outperform their state secondary school counterparts regardless of ability. Future UK policies should focus on reducing social inequality in education to ensure that all children are offered the same life chances regardless of background.

## Introduction

Social inequalities in education have long been documented in great detail in many modern societies, with social forces acting directly to increase or maintain them through lack of material resources or non-alleviation of developmental problems (McLoyd, 1998), or indirectly through parental attitudes to education, to children and parents’ ability to help (Phillips, Brooks-Gunn, Duncan, Klebanov, & Crane, 1998). In the UK children from family backgrounds characterised by high socio-economic position (SEP) outperform their lower SEP counterparts (Bynner & Joshi, 2002). Research into social inequalities in education has demonstrated that not only are disadvantaged children far less likely to become high academic attainers at any stage (Crawford, Macmillan, & Vignoles, 2014) but that high-attaining socio-economically disadvantaged children are academically overtaken by their average-attaining, more economically advantaged peers (Goodman & Gregg, 2010). In the UK and the US these differences appear as early as pre-school but there is conflicting evidence on how developmental trajectories diverge (Centre for Market and Public Organisation [CMPO], 2006; Cunha, Heckman, Lochner, & Masterov, 2006; Feinstein, 2003; Goodman & Gregg, 2010; Tomlinson, 2013).

This paper concerns just the UK but its findings may be more widely applicable. In the UK children from the most deprived quintile of families are 3 times less likely to achieve highly in both Maths and English at age 7 compared to those from the least deprived quintile, rising to three and a half times less likely at age 11, and over 4 times less likely when they finish school at age 16 (Crawford et al., 2014). In 2011 data from the Department for Education (DFE, 2012) revealed that of children eligible for free school meals (FSM), a proxy for low income, only 35% attained 5 or more A*–C grades (including English and Maths) compared to 62% of non-FSM children. Social differences in attainment not only exist, but are greater than gender or ethnicity differences; around six times larger than gender differences and three times larger than ethnicity differences (Strand, 2011).

Figures on changes in pass rates in recent years and the great inequalities outlined in the paragraph above imply that great changes are possible in a relatively short period of time; if an extra one in five of the poorest children in Britain can secure good exam passes with focussed national priority effort (Dorling, 2015a) then it is conceivable that longer term national effort will be able to further decrease (or at least stabilise) social inequalities in education. Similar sudden improvements were seen in the past when, for instance, compulsory elementary schooling was introduced in various countries and literacy rates improved markedly because far more children were being taught to read (Dorling, 2015b).

Social inequalities in education are further complicated by the competitive schools market and the existence of fee-paying institutions alongside state schools in the UK that are able to use social class and other demographic factors as filters (Tomlinson, 2014). Elite school attendance that is generally reserved for children from high SEP families has independent beneficial effects on long term life outcomes above individual educational achievements (Clark & Del Bono, 2014). It therefore appears that the education system within the UK is failing socio-economically disadvantaged children by not allowing them to even reach their expected educational level based upon their early childhood achievements. Only some 38% of children receiving free school meals due to their parents’ poverty achieve good exam passes at age 16; a rapid improvement on 18% only eight years earlier (Social Mobility and Child Poverty Commission, 2014). It is possible that grade inflation over this time may account for some of this rise as non-FSM students also saw an increase from 46% to 65% (Strand, 2015), but this would need to have been rapid to account for such a large change and regardless represents a disproportionate improvement in FSM student attainment as compared to other students who should also have seen grade rises if there had been general inflation. If this improvement is possible what else might be possible; especially if such high levels of poverty were not tolerated? Here we do not consider important wider questions such as whether there is more to achievement than gaining A* to C grades at GCSE and A's at A level.

In addition to these social processes, academic attainment is also known to be heavily influenced by genetic and biological factors (Davies, Hermani, Timpson, Windmeijer, & Davey Smith, 2015). This is most clearly the case for children born with organic brain damage, or having other severe learning difficulties. Beyond this there is an active debate over the extent to which genetic endowment and inherited abilities allow some children to start their schooling experience ‘ahead’ of others in a similar way. Intelligence correlation as reflected by particular ability tests is strongly associated between parents and their children, and has a strong positive correlation with success in a range of academic subjects (Deary, Strand, Smith, & Fernandes, 2007). Despite a large body of evidence of the genetic/biological influences on educational attainment there is also strong evidence that outcomes are environmentally (i.e. socially) contingent (Branigan, McCallum, & Freese, 2013). The jump from just 18–38% of the poorest children in state funded schools in England passing 5 or more GCSEs at grade A* to C is indicative of such social influences.

Due to dataset limitations and the highly complex and changing nature of examination and grades many studies have been unable to include reliable measurements of early childhood tests of cognitive ability, SEP, and educational attainment, leading to potentially biased findings. To our knowledge no work on a general cohort of the recent UK population has been conducted examining socio-economic disparities in the relationship between cognitive ability and educational attainment. Work has been conducted on previous UK cohorts born in 1958 (Feinstein, 2000) and 1970 (Feinstein, 2003) demonstrating socio-economic inequalities in educational attainment but these findings are based on cohorts that passed through school three to four decades ago at a time when UK social inequalities were far different to those today (Dorling, 2015b). We therefore seek to contribute to this literature by examining educational outcomes across a broad range of indicators of SEP – while accounting for cognitive ability – in a recent cohort in order to determine the extent of social inequalities in compulsory and post-compulsory UK educational attainment.

## Methods

### Study population

Participants were children from the Avon Longitudinal Study of Parents and Children (ALSPAC). Pregnant women were eligible to enrol if they had an expected date of delivery between April 1991 and December 1992 and were resident in the (former) Avon Health Authority area in South West England (for full details of the cohort profile and study design see Boyd et al*.* (2013) and Fraser et al*.* (2013)). Figure S1 in the Supplementary material shows the available analytical sample for our study and causes of attrition. From the core sample of 14,676 children 4049 have full data on educational outcomes, cognitive ability and maternally reported SEP. Paternal questionnaire responses are lower than maternal questionnaire responses in ALSPAC so the analysis utilising paternally reported income utilises a reduced sample of 1768. This approach allows us to examine differences between incomes at different ages without restricting our sample size for all analyses. The ALSPAC cohort is largely representative of the UK population when compared with 1991 Census data; however there is under representation in ethnic minorities, single parent families, and those living in rented accommodation. Ethical approval for the study was obtained from the ALSPAC Ethics and Law Committee and the Local Research Ethics Committees. Please note that the study website contains details of all the data that is available through a fully searchable data dictionary (ALSPAC data dictionary available at http://www.bris.ac.uk/alspac/researchers/data-access/data-dictionary/).

### Exposures

A range of exposure variables were tested to examine the relationship between different aspects of family social status and educational attainment. These include highest maternal education at pregnancy (categorised as certificate of secondary education (CSE)/none/Vocational, O-level [exams taken at completion of compulsory school attendance], A-level [exams taken in post-compulsory schooling at age 18], and university degree or above); a family adversity index measuring multiple aspects of household deprivation (see supplementary material – the two least deprived categories were combined due to low numbers); highest parental Social Class based on Occupation (due to low numbers social classes I (Professional occupations) and II (Managerial and technical occupations) were combined together, and IV (Partly skilled occupations) and V (Unskilled occupations) combined); and income reported by mothers at age 7 and mothers’ partner at age 11.

### Outcomes

Educational attainment data was taken from the UK National Pupil Database (NPD) at Key Stage 4 (GCSE) examinations and Key Stage 5 (A-level) then linked to ALSPAC children whom had given consent for data linkage. GCSE examinations are sat during the 11th year of compulsory schooling when children are aged 15/16 (years 2007–2009 for the ALSPAC cohort) and A-levels are sat during the second year of post-compulsory schooling when children are aged 17/18 (years 2009–2011 for the ALSPAC cohort). We used Maths and English at GCSE as these are the two core subjects most widely required for participation in further education and the labour market. . Due to the wide range of subjects available at A-level and the resulting data confidentiality issues, A-level subject grades were averaged then grouped into categories representing different grade combinations (see Supplementary material Table S1).

### Cognitive ability

Cognitive ability was assessed at the age eight clinic visit using the Wechsler Intelligence Scale for Children (WISC) to assess cognitive function from verbal, performance, and digit span tests (Wechsler, 1992). The WISC test was administered by members of the ALSPAC psychology team and overseen by an expert in psychometric testing, and at the time of the clinic was the most widely used individual ability test worldwide. In order to reduce the likelihood of tiredness amongst children when performing the test a short form of the measure was utilised where alternate items were used for all subsets, with the exception of the coding subtest which was administered in its full form. Short form tests are considered to have high reliability (Crawford, Anderson, Rankin, & MacDonald, 2010) and have previously been used successfully in studies (Finch & Childress, 1975; Stricker, Merbaum, & Tangeman, 1968). In addition, the ALSPAC measures show the same Genome-wide Complex Trait Analysis heritability as full form measures in other cohorts (Benyamin et al., 2014) and utilise subtests with reliability ranging from 0.70 to 0.96. Raw scores were recalculated to be comparable to those that would have been obtained had the full test been administered then age-scaled to give total scores for the performance and verbal scales, and a total overall score. Our analyses focussing on high ability children uses the definition of high ability as being in the top 10% of the cognitive ability distribution.

### Statistical analysis

First we use ANOVA to determine the predictive ability of age eight cognitive ability on attainment at GCSE and A-level, with results presented in graphical form for ease of interpretation. We then proceed to examine differences in attainment between children by socio-economic background, presenting differences in the proportions of high ability children attaining ‘good’ grades (defined as B + in English and Maths at GCSE, and AAA + at A-level; see Supplementary material for discussion of grade boundary choice) from differing socio-economic backgrounds. These are complemented by results from a multivariate regression analysis on the full sample (we omit income at 11 due to sample size issues). Lastly we demonstrate attainment differences between children attending state and fee-paying schools. All statistical analyses were performed using STATA v 13 (StataCorp, College Station, TX, USA).

There has been considerable recent debate over the statistical phenomenon of regression towards the mean (RTM) when modelling the progress trajectories of high attaining, socio-economically disadvantaged groups. Jerrim and Vignoles (2013) point out that the convergence of trajectories is not entirely due to the disproportionate progress made by high SEP children but because of corrections to high measurement error in baseline cognitive ability tests that even out over time with subsequent measures. They provide an RTM calibrated analysis of cognitive ability over time from a separate cohort and while their results demonstrate that low ability high SEP children do not catch up to high ability low SEP children as fast as previously thought, there is still a considerable narrowing of trajectories that cannot be explained by RTM alone, as has been found in other studies (Crawford et al., 2014). Because we utilise only one measure of cognitive ability and do not examine trajectories of ability per se, it is measurement error rather than RTM that is relevant to our paper. We therefore use two approaches to account for measurement error in the WISC test as additional analyses and present these in the Supplementary material.

## Results

### Participant characteristics

Table 1 displays the characteristics of participants in our main sample. Just under half (*n* = 1951; 48.18%) of participants attained a B or above in Maths and just over half (*n* = 2099; 51.84%) attained a B or above in English. Gender differences were observed for English (girls B + attainment 61.19%; boys B + attainment 41.77%) but not Maths (girls B + attainment 48.10%; boys B + attainment 48.28%) (data not displayed in Table 1).

**Table 1.** Participant characteristics.

		Excluded from main sample *n* (%)	Included in main sample *n* (%)
Sex	Male	5690 (52.63)	1949 (48.14)
*χ*^2^ = 23.79, *p* < .001	Female	5122 (47.37)	2100 (51.86)
Household deprivation score	1 – Least deprived	4420 (38.79)	1264 (31.22)
*χ*^2^ = 145.67, *p* < .001	2	2461 (21.6)	1106 (27.32)
	3	1743 (15.29)	758 (18.72)
	4	1071 (9.40)	455 (11.24)
	5 – Most deprived	1701 (14.93)	466 (11.51)
Maternal education	Degree	1001 (11.85)	609 (15.04)
*χ*^2^ = 401.13, *p* < .001	A level	1643 (19.44)	1161 (28.67)
	O level	2835 (33.55)	1494 (36.90)
	CSE/Vocational	2971 (35.16)	785 (19.39)
Social class	I and II	1854 (24.7)	1156 (28.55)
*χ*^2^ = 92.95, *p* < .001	III-NM	1750 (23.31)	1140 (28.16)
	III-M	2243 (29.88)	1099 (27.14)
	IV and V	1659 (22.1)	654 (16.15)
Family income at 7	£400+	1464 (41.82)	1858 (45.89)
*χ*^2^ = 94.65, *p* < .001	£300–£399	718 (20.51)	967 (23.88)
	£200–£299	634 (18.11)	754 (18.62)
	<£199	685 (19.57)	470 (11.61)
Scored B or above in Maths	No	5238 (72.11)	2098 (51.82)
*χ*^2^ = 469.69, *p* < .001	Yes	2026 (27.89)	1951 (48.18)
Scored B or above in English	No	4925 (66.94)	1950 (48.16)
*χ*^2^ = 384.81, *p* < .001	Yes	2432 (33.06)	2099 (51.84)
Family income at 11	£800+	435 (26.92)	315 (17.82)
*χ*^2^ = 59.23, *p* < .001	£480–£799	583 (36.08)	797 (45.08)
	£290–£479	427 (26.42)	521 (29.47)
	<£289	171 (10.58)	135 (7.64)

These results are consistent with the latest national results for England. They are worth reporting because of the changes in recent years that suggest rapidly changing social factors matter: We know that there will not have been a sudden change in the nature of girls and boys or in what poorer and richer children inherit biologically from their parents. Thus when there are rapid divergences in the exam successes of girls and boys in the space of a few years, or of poorer children suddenly achieving much better exam results, as illustrated above with free school meals, then the importance of social and culture factors is made clearer. The gap between the percentage of girls and boys achieving 5 or more GCSEs at grade A* to C or equivalent including English and Maths has widened by 2.8 percentage points since 2008/2009 to 10.1 percentage points in 2012/2013, with 65.7% of girls achieving this indicator compared to 55.6% of boys (DFE, 2014).

### Education attainment

**Figure 1.**
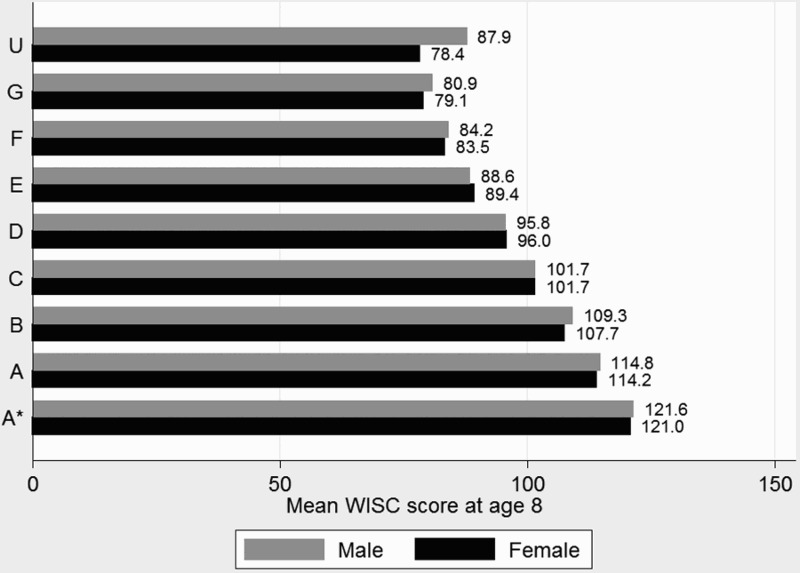
Mean WISC scores at 8 years by GCSE Maths grade.

**Figure 2.**
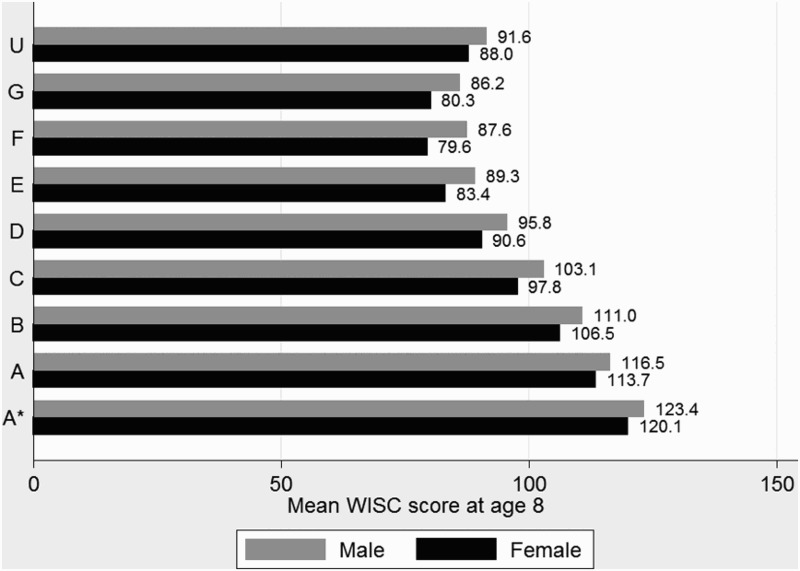
Mean WISC scores at 8 years by GCSE English grade.

**Figure 3.**
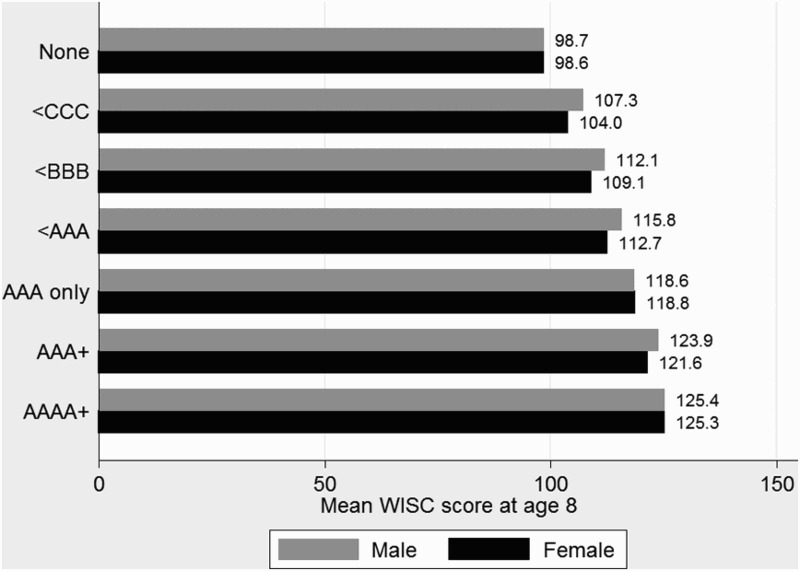
Mean WISC scores at 8 years by A-level grade groups.

Figures 1–3 show the most simple relationship between children's cognitive ability at age 8 and educational attainment at age 16 with boys and girls seperately grouped according to their attainment in GCSE Maths (Figure 1) and English (Figure 2) with corresponding group mean WISC scores. Both figures show the predictive power of cognitive ability on GCSE attainment; a monotonic improvement in WISC score at age eight is observed for every grade improvement from grade G, with grade F on English for girls being the only exception. Gender differences in WISC score by attainment categories are larger for English than Maths, but for any group of children there will still be a wide variation in outcomes at age 16 regardless of ability at age 8.

Age 18 A-level results (Figure 3) show the same linear pattern as GCSE results but with an even stronger relationship; each increase in grade above the baseline group (those who did not attain A-levels) is associated with a higher WISC score for both sexes. Small gender differences exist in the lower categories of attainment but these decrease in the higher categories. In short, cognitive ability as measured by the WISC predicts educational outcomes extremely well.

### Socio-economic inequalities in educational attainment

Table 2 displays the proportion of age eight high ability children who attained grade B or above in GCSE Maths and English by a range of SEP measures. Patterns of attainment were consistently linear across all measures whereby children from lower SEP backgrounds were disproportionately less likely to attain a B or above in GCSE Maths or English than children from higher SEP backgrounds, despite similar high cognitive ability as determined by the WISC test. The strength of effect varied across SEP measures being strongest on maternal education and partner reported income for both subjects. The results indicate that children who come from more deprived family backgrounds are less likely to fulfil their intellectual potential when it comes to GCSE attainment. It is worth noting that socio-economic patterning of attainment persisted when using C + and A + grade boundary cut-offs instead of B +.

**Table 2.** B + attainment in GCSE Maths and English for children in top 10% of WISC by SES measures.

		Scored B or above in Maths		Scored B or above in English	
		No	Yes	No	Yes
Household deprivation score	1 – Least deprived	27 (10.63)	227 (89.37)	28 (11.02)	226 (88.98)
	2	28 (14.29)	168 (85.71)	32 (16.33)	164 (83.67)
	3	19 (12.93)	128 (87.07)	23 (15.65)	124 (84.35)
	4	14 (18.67)	61 (81.33)	12 (16.00)	63 (84.00)
	5 – Most deprived	20 (30.30)	46 (69.70)	23 (34.85)	43 (65.15)
Maternal education	Degree	14 (5.71)	231 (94.29)	15 (6.12)	230 (93.88)
	A level	34 (13.65)	215 (86.35)	36 (14.46)	213 (85.54)
	O level	40 (21.39)	147 (78.61)	43 (22.99)	144 (77.01)
	CSE/Vocational	20 (35.09)	37 (64.91)	24 (42.11)	33 (57.89)
Social class	I and II	30 (8.60)	319 (91.40)	35 (10.03)	314 (89.97)
	III-NM	28 (13.93)	173 (86.07)	30 (14.93)	171 (85.07)
	III-M	38 (27.74)	99 (72.26)	41 (29.93)	96 (70.07)
	IV and V	12 (23.53)	39 (76.47)	12 (23.53)	39 (76.47)
Family income at 7	£400+	41 (9.26)	402 (90.74)	47 (10.61)	396 (89.39)
	£300–£399	28 (19.18)	118 (80.82)	34 (23.29)	112 (76.71)
	£200–£299	25 (26.32)	70 (73.68)	22 (23.16)	73 (76.84)
	<£199	14 (25.93)	40 (74.07)	15 (27.78)	39 (72.22)
Family income at 11	£800+	6 (5.83)	97 (94.17)	7 (6.80)	96 (93.20)
	£480–£799	22 (11.46)	170 (88.54)	22 (11.46)	170 (88.54)
	£290–£479	17 (26.15)	48 (73.85)	14 (21.54)	51 (78.46)
	<£289	6 (33.33)	12 (66.67)	8 (44.44)	10 (55.56)

Table 3 displays the proportion of high ability children attaining high grades at A-level (AAA or above) across a range of SEP measures. Similar to the GCSE results in Table 2, children from lower SEP backgrounds were disproportionately less likely to attain high A-level grades than children from high SEP backgrounds on every indicator despite similar cognitive ability. Associations were strongest for income and maternal education, but these results should be interpreted with caution due to low cell counts. As with GCSE results, this socio-economic patterning of attainment persisted across different grade boundary cut-offs.

**Table 3.** AAA + attainment in A-levels for children in top 10% of WISC by SES measures.

		Attained AAA + in A-levels	
		No	Yes
Household deprivation score	1 – Least deprived	185 (76.13)	58 (23.87)
	2	146 (77.66)	42 (22.34)
	3	105 (77.78)	30 (22.22)
	4	67 (90.54)	7 (9.46)
	5 – Most deprived	46 (79.31)	12 (20.69)
Maternal education	Degree	157 (65.97)	81 (34.03)
	A level	198 (81.82)	44 (18.18)
	O level	153 (87.93)	21 (12.07)
	CSE/Vocational	41 (93.18)	3 (6.82)
Social class	I and II	247 (73.29)	90 (26.71)
	III-NM	154 (80.21)	38 (19.79)
	III-M	106 (85.48)	18 (14.52)
	IV and V	42 (93.33)	3 (6.67)
Family income at 7	£400+	308 (72.81)	115 (27.19)
	£300–£399	116 (82.86)	24 (17.14)
	£200–£299	78 (89.66)	9 (10.34)
	<£199	47 (97.92)	1 (2.08)
Family income at 11	£800+	65 (65.66)	34 (34.34)
	£480–£799	144 (77.42)	42 (22.58)
	£290–£479	52 (83.87)	10 (16.13)
	<£289	14 (93.33)	1 (6.67)

As there is a possibility that our high ability group may suffer from a disproportionate amount of measurement error to the full sample, we ran full regression models with GCSE Maths, GCSE English, and A-level grade as outcome variables, and SEP variables as predictor variables. The results of these are presented both unadjusted and adjusted for age eight cognitive ability in Table 4. The results display that for every educational outcome socio-economic disparities in attainment exist for each measure of SEP, with associations strongest for maternal education and weakest for household deprivation. Family SEP predicted A-level attainment better than GCSE English and GCSE Maths attainment, which were predicted broadly similar. After adjustment for cognitive ability associations are attenuated but remain robust for every tested measure of SEP, lending weight to our initial analysis examining only high ability children. In short, socio-economic inequalities in educational attainment exist between children who had the same measured cognitive ability at age eight, regardless of whether they are deemed high ability or not.

**Table 4.** Regression analysis of SEP measures on educational attainment, unadjusted and adjusted for WISC score.

	GCSE Maths				GCSE English				A-level			
	Unadjusted		Adjusted		Unadjusted		Adjusted		Unadjusted		Adjusted	
Household deprivation	Coef. (95% CI)	*p*	Coef. (95% CI)	*p*	Coef. (95% CI)	*p*	Coef. (95% CI)	*p*	Coef. (95% CI)	*p*	Coef. (95% CI)	*p*
2	−0.05 (−0.17 to 0.06)	.361	−0.05 (−0.15 to 0.05)	.300	−0.10 (−0.20 to 0.00)	.047	−0.10 (−0.19 to −0.01)	.029	−0.31 (−0.65 to 0.03)	.073	−0.30 (−0.62 to 0.01)	.060
3	−0.06 (−0.19 to 0.07)	.335	−0.08 (−0.18 to 0.03)	.172	−0.19 (−0.30 to −0.08)	.001	−0.20 (−0.30 to −0.10)	<.001	−0.73 (−1.11 to −0.35)	<.001	−0.76 (−1.11 to −0.41)	<.001
4	−0.29 (−0.44 to −0.13)	<.001	−0.24 (−0.37 to −0.11)	<.001	−0.16 (−0.29 to −0.03)	.015	−0.13 (−0.25 to −0.01)	.033	−0.92 (−1.37 to −0.46)	<.001	−0.81 (−1.23 to −0.39)	<.001
5 – most deprived	−0.37 (−0.53 to −0.22)	<.001	−0.36 (−0.50 to −0.23)	<.001	−0.25 (−0.39 to −0.12)	<.001	−0.25 (−0.37 to −0.13)	<.001	−0.80 (−1.27 to −0.34)	.001	−0.79 (−1.22 to −0.36)	<.001
Maternal education												
A-level	−0.60 (−0.75 to −0.46)	<.001	−0.26 (−0.39 to −0.14)	<.001	−0.45 (−0.57 to −0.33)	<.001	−0.21 (−0.32 to −0.10)	<.001	−2.44 (−2.87 to −2.01)	<.001	−1.73 (−2.13 to −1.34)	<.001
O-level	−0.96 (−1.11 to −0.81)	<.001	−0.45 (−0.58 to −0.32)	<.001	−0.80 (−0.93 to −0.68)	<.001	−0.44 (−0.56 to −0.32)	<.001	−3.98 (−4.42 to −3.53)	<.001	−2.91 (−3.33 to −2.50)	<.001
CSE/vocational	−1.42 (−1.59 to −1.25)	<.001	−0.70 (−0.85 to −0.55)	<.001	−1.15 (−1.30 to −1.01)	<.001	−0.64 (−0.77 to −0.50)	<.001	−4.87 (−5.37 to −4.36)	<.001	−3.36 (−3.84 to −2.88)	<.001
Social class												
III-NM	−0.08 (−0.21 to 0.04)	.189	−0.01 (−0.11 to 0.10)	.923	−0.04 (−0.15 to 0.06)	.418	0.01 (−0.08 to 0.11)	.814	−0.66 (−1.03 to −0.30)	<.001	−0.5 (−0.84 to −0.16)	<.001
III-M	−0.44 (−0.58 to −0.31)	<.001	−0.24 (−0.35 to −0.13)	<.001	−0.42 (−0.54 to −0.31)	<.001	−0.28 (−0.38 to −0.18)	<.001	−1.79 (−2.18 to −1.40)	<.001	−1.36 (−1.73 to −1.00)	<.001
IV & V	−0.69 (−0.84 to −0.54)	<.001	−0.37 (−0.50 to −0.24)	<.001	−0.57 (−0.70 to −0.44)	<.001	−0.34 (−0.46 to −0.22)	<.001	−1.97 (−2.41 to −1.52)	<.001	−1.29 (−1.71 to −0.87)	<.001
Family income at 7												
£300–399	−0.12 (−0.24 to −0.01)	.032	−0.06 (−0.16 to 0.04)	.216	−0.12 (−0.22 to −0.02)	.017	−0.07 (−0.16 to 0.01)	.098	−0.33 (−0.67 to 0.00)	.053	−0.20 (−0.51 to 0.11)	.210
£200–299	−0.30 (−0.43 to −0.17)	<.001	−0.20 (−0.30 to −0.09)	<.001	−0.24 (−0.34 to −0.13)	<.001	−0.16 (−0.26 to −0.07)	.001	−0.98 (−1.36 to −0.61)	<.001	−0.77 (−1.12 to −0.42)	<.001
<£199	−0.51 (−0.66 to −0.35)	<.001	−0.33 (−0.46 to −0.20)	<.001	−0.45 (−0.58 to −0.32)	<.001	−0.33 (−0.45 to −0.21)	<.001	−1.46 (−1.92 to −1.00)	<.001	−1.11 (−1.53 to −0.68)	<.001
WISC			0.05 (0.05 to 0.05)	<.001			0.04 (0.03 to 0.04)	<.001			0.11 (0.10 to 0.12)	<.001

Notes: Coef., regression coefficient; 95% CI, 95% confidence interval; *p*, *p* value; CSE, certificate of secondary education; III-NM, Social class III (non-manual); III-M, Social class III (manual); IV, Social class IV; V, Social class V; WISC, WISC test cognitive score.

Given concerns of measurement error we ran two additional sets of analyses which are presented in the Supplementary material (Tables S8 and S9). The first of these utilises an instrumental variables approach and uses cognitive ability measured at age four for a small subsample of ALSPAC participants (*n* = 463) as an instrument for cognitive ability at age eight. The second utilises an error in variables regression analysis which allows results to be tested with varying levels of variable reliability. The results of these analyses, presented and discussed in more detail in the Supplementary material indicate that our main results are robust to WISC measurement error. Further additional analyses were conducted to determine if our results could be influenced by differences in maternal cognitive ability (*n* = 1676) (Tables S10–S15 in Supplementary material). Results show that maternal cognitive ability strongly predicts child educational outcomes and remains even after adjustment for child cognitive ability and it is therefore possible that our results may be influenced in part by the endogenous link between certain SEP measures and maternal cognitive ability.

### School type differences on educational attainment

**Figure 4.**
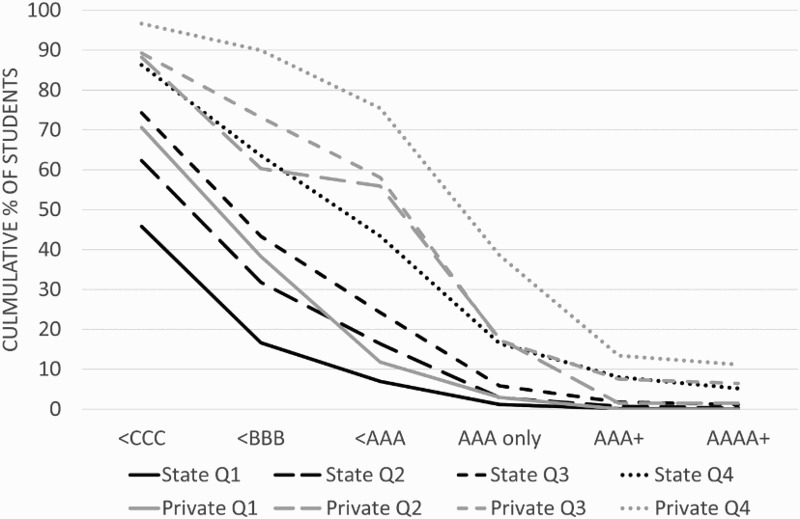
Cumulative proportions of A-level grades for all low and high ability children in state and fee-paying secondary schools. Q1, Quartile 1 (low ability children); Q2, Quartile 2; Q3, Quartile 3; Q4, Quartile 4 (high ability children).

The easiest way to see the effect of school type is to compare the progress of children attending different types of school establishment. Figure 4 displays the cumulative proportions of A-level attainment for all children separated into quartiles of cognitive ability amongst non-selective state schools and private fee-paying schools.^1^ The results show differences in both entry to, and performance in A-levels between school types. A lower proportion of children at fee-paying schools fail to go on and attain any A-levels than children of similar cognitive ability at state schools, and those that do study A-levels outperform their state schooled counterparts in every quartile of cognitive ability. This relationship is so strong that children in the bottom quartile of cognitive ability attending fee-paying schools perform broadly similar in A-levels to children in state schools who are in the quartile above them for cognitive ability, and children in all other quartiles attending fee-paying schools perform at least as well as children in the highest quartile of ability who attend state schools. Results from a linear regression analysis presented in Table S17 suggest that for an average child attending a fee-paying school is comparable to having a WISC score 19 points higher in terms of A-level outcomes. These results imply that children at fee-paying schools hold a considerable advantage over similar cognitive ability children in state schools.

## Discussion

These findings indicate considerable social inequality in educational attainment in the UK even after accounting for child cognitive ability. There is evidence that high ability children who come from more socio-economically deprived family backgrounds are less likely to fulfil their cognitive potential regarding GCSE attainment by up to a factor of 1.6 and A-level attainment by up to a factor of 7.8. Here ‘cognitive potential’ means what educational attainment we would have expected of a similar child who scored as well at age eight but came from a socio-economically better off home. Maternal education remained the strongest determinant of social inequalities in attainment among high ability children at both GCSE and A-level, followed by family income. Our results remain when considering a wide range of indicators of SEP together and conform to previous findings that non-deprived, high achieving students in the UK are more likely to maintain high levels in education than deprived, high achieving students (Crawford et al., 2014). While we only had WISC measured at one time point for the vast majority of our sample and were therefore unable to account for RTM which may have introduced bias to our analysis in Tables 2 and 3 (see Supplementary material for a detailed explanation), our sensitivity analyses indicated that our main results in Table 4 should not be biased by measurement error in the WISC test. However, these findings should still be interpreted with a certain degree of caution because observed SEP differences in educational outcome may in part be influenced by differences in maternal cognitive ability.

Our findings that students at fee-paying secondary schools perform better, especially if they performed poorly at age eight, as compared to those at non-selective state secondary schools is consistent with recent research on a large, nationally representative cohort of UK children (Crawford et al., 2014). Our results build on this previous knowledge to show that while some of this is due to fee-paying schools in general capturing higher ability students, low ability students in fee-paying schools are equally as likely to study for A-levels and receive high grades compared to higher ability children in state secondary schools. Given that entrance to non-selective fee-paying schools in the UK is almost entirely dependent on family financial power, the observed differences in outcomes of similar ability children between school types is indicative of social inequality. The use of highly restrictive fee-paying institutions and their ability to improve the educational outcomes of lower ability students serves to maintain and increase social divisions in UK education; it permits ‘class based opportunism that competitively seeks education to gain and sustain privilege’ (Tomlinson, 2014, p. 120). It should be noted that the spending per child is higher in fee-paying schools than state schools and therefore the SEP differences that we observe may not solely be due to family background. It is also possible that parents who send their children to fee-paying schools consider exam results more important than parents who send their children to state schools, and that this difference in values may explain some of the differences that we observe, however we were unable to measure this.

The data presented suggest that it would be more socially equitable if resources for children from disadvantaged backgrounds were improved in order to bring their outcomes in line with those of children from more advantaged backgrounds (Blau & Currie, 2006) and ensure greater social equality in education. Education, after all, should not permit certain individuals to gain unfair advantages at the expense of others (Tomlinson, 2014). The socio-economic discrepancies that our results reveal provide evidence that family SEP may play a strong role in the ability of children to achieve the academic potential that can be predicted from how well they score at age eight. While the use of genetic variables was beyond the scope of this paper, our use of the thoroughly administered WISC test provided a robust indicator of a combination of inherent cognitive ability and already learnt behaviour by age eight combined.

Our use of educational linkage data meant that school type data and grade outcomes were accurate and not subject to reporting bias. The use of the clinically assessed WISC test provided accurate measures of children's cognitive ability at age eight, and due to the timing of the test before subject-specific teaching at secondary school (Deary et al., 2007), the age of participants at the time of testing (Hopkins & Bracht, 1975; Jensen, 1980) and the controlled conditions in which the cognitive ability tests were sat WISC measurement error was minimised. Studies with repeat measures from the WISC test have demonstrated low measurement error and high test-retest reliability (Kamphaus, 2005) and our instrumental variables and error in variables regression sensitivity analyses demonstrate that our results are robust to a highly conservative level of measurement error. However, a number of limitations exist in our study. Firstly, missing data for family SEP was a problem, particularly for partner reported income. Due to biases in cohort attrition and the likelihood of data being missing not at random it is therefore conceivable that missing data may have led to a disproportionate exclusion of low SEP families and therefore biased results. However, because this paper examines the association between WISC and educational outcomes and is not intended to present data on average levels or the distribution of factors across the population, non-representativeness will not invalidate our findings unless complex forms of associational drop out exist (see Rothman, Gallacher, and Hatch (2013) for a discussion the issue of representativeness). Secondly, our cohort is largely geographically defined and therefore may not be representative of all UK children, although our participant characteristics indicate that this is not the case in terms of educational outcomes. Thirdly, as we only have cognitive ability test results from one time point we were unable to assess the degree to which test score measurement error varied across SEP groups. There is no substantive reason to believe that measurement error should have been disproportionately greater for children from lower SEP groups than those from higher SEP groups at this age, however, the fact that maternal cognitive ability was predictive of child education outcomes independent of child's own cognitive ability, and that certain SEP measures such as maternal education are endogenous of cognitive ability makes it important that we acknowledge this.

## Conclusions

Differences exist in mean cognitive ability between groups of children by educational attainment at age sixteen and eighteen whereby higher attaining groups have higher mean cognitive ability. However social inequalities in educational ability persist above this relationship; low SEP children systematically underperform their high SEP peers regardless of cognitive ability. In addition to family background characteristics school effects operate to add to this inequality as children in fee-paying secondary schools outperform their state secondary school counterparts regardless of ability.

## Supplementary Material

Morris Supplemental Data.docx

## Note

1 Table S16 in the Supplementary material displays individual cell numbers and percentages.
