# Supplementary material for: How well can we predict educational outcomes? Examining the roles of cognitive ability and social position in educational attainment
Source: Contemp Soc Sci. 2016 Feb 23;11(2-3):154–68. doi: 10.1080/21582041.2016.1138502 (PMC5283177; doi:10.1080/21582041.2016.1138502)
Supplement: Morris Supplemental Data.docx [file rsoc_a_1138502_sm0030.docx]

**Supplementary Appendix**

Supplement to: Morris T, Dorling D, Davey Smith G. **How well can we predict educational outcomes? Examining the roles of cognitive ability and social position in educational attainment.**

***Components of family adversity index***

The ALSPAC family adversity index in pregnancy (1 point scored for each applicable item).

Demographic variables

Mother aged <20 years at delivery of study child

Mother aged <17 years at delivery of first child

Housing

Homeless

Number of people per room >1

Shared use of bath or shower

No running hot water

No indoor toilet

No kitchen

Mould present

Leaking roof

Rodents or cockroaches present

Education

Mother has no qualifications

Partner has no qualifications

Poor financial circumstances

Derived from 5 questions about difficulty affording food, clothing, heating, housing, and items for the baby

Relationship with partner

No partner

No intimate bond

No affection

High aggression

Physically cruel

Emotionally cruel

No emotional support

No practical support

Afraid partner might leave

Social network

Not able to share feelings

No one to discuss problems with

No friends to borrow money from in times of need

No relations to borrow money from in times of need

Maternal emotional status

High anxiety score

High depression score

Has attempted suicide

Substance abuse

Uses hard drugs

Has alcohol problem

Partner has alcohol problem

Drinks >2 units alcohol per day

Partner drinks >2 units alcohol per day

Crime

In trouble with police

Convicted of an offence

Partner in trouble with police

***Figure S1: Flowchart describing ALSPAC cohort attrition in analytical sample***

Complete cases: data on Linkage, WISC, and SEP available

(n = 4 049)

(n = 4 250)

Children enrolled in ALSPAC

(n = 15 458)

Singletons and twins alive at 1 year

(n = 14 691)

Linkage data available

(n = 10 549)

WISC data available

(n = 5 702)

Missing data on SEP

(n = 1 653)

Not alive at 1 year; triplets & quadruplets (n = 767)

Missing Linkage data

(n = 4 142)

Missing WISC data

(n = 4847)

***Table S1: A-level grade combination groups.***

| Group | Possible grade combinations |
| --- | --- |
| 1 | EEE |
| 2 | DEE |
| 3 | DDE/CEE |
| 4 | DDD/CDE/BEE |
| 5 | CDD/BDE/AEE/CCE |
| 6 | BDD/ADE/BCE/CCD |
| 7 | CCC/ACE/ADD/BCD |
| 8 | ABE/ACD/BBD/BCC |
| 9 | AAE/ABD/ACC/BBC |
| 10 | AAD/ABC/BBB |
| 11 | AAC/ABB |
| 12 | AAB |
| 13 | AAA |
| 14 | AAA+1 |
| 15 | AAAA |
| 16 | AAAA+1 |

***Justification for B+ GCSE and AAA+ A-level grade boundary***

When making the decision to select a grade boundary for our analysis in Table 2 of our main text a number of limiting factors had to be taken into account. We were unable to categorise a ‘good’ GCSE grade as C or above (as we had originally intended to do) due to low numbers of children in the top 10% of the WISC distribution scoring below a C in their GCSE examinations. Therefore, the decision was made to raise the grade boundary to the next highest grade, B. As is displayed in Table 2, sufficient numbers of participants in our analytical sample were in the top 10% of the WISC distribution and attained grade C or below in each of our SEP measures. In the interest of determining the robustness of our results the analysis for Table 2 was also run on each grade boundary of C+, A+, and A*+ for GCSE English and Maths. These results, not presented here but available from the authors, consistently show the same socioeconomic patterning of attainment as those for B+.

For A-level the decision was taken to use “AAA+” as the high attainment group as this is what is required by the very top UK universities for entry into their courses. Similar to GCSE grade boundaries though, the broad socioeconomic patterning that is observed in Table 3 is repeated no matter whether a CCC, BBB, or AAA boundary is selected. These results are also available from the authors upon request.

***Table S2: Grouped WISC score for boys Maths GCSE attainment***

| Grade attained in GCSE Maths | N | Coef. | 95% Conf. Interval | p value |
| --- | --- | --- | --- | --- |
| U* | 23 | 0 |  |  |
| G | 61 | -6.979 | (-13.088 to -0.869) | 0.025 |
| F | 107 | -3.698 | (-9.437 to 2.041) | 0.206 |
| E | 250 | 0.679 | (-4.762 to 6.120) | 0.807 |
| D | 411 | 7.866 | (2.515 to 13.216) | 0.004 |
| C | 773 | 13.788 | (8.505 to 19.071) | <0.001 |
| B | 630 | 21.427 | (16.126 to 26.727) | <0.001 |
| A | 440 | 26.885 | (21.544 to 32.225) | <0.001 |
| A* | 216 | 33.689 | (28.212 to 39.165) | <0.001 |
| Constant | 2911 | 87.913 | (82.707 to 93.119) | <0.001 |

***Table S3: Grouped WISC score for girls Maths GCSE attainment***

| Grade attained in GCSE Maths | N | Coef. | 95% Conf. Interval | p value |
| --- | --- | --- | --- | --- |
| U* | 20 | 0 |  |  |
| G | 47 | 0.749 | (-5.536 to 7.034) | 0.815 |
| F | 130 | 5.138 | (-0.516 to 10.793) | 0.075 |
| E | 252 | 11.025 | (5.556 to 16.493) | <0.001 |
| D | 418 | 17.552 | (12.164 to 22.941) | <0.001 |
| C | 883 | 23.299 | (17.975 to 28.622) | <0.001 |
| B | 646 | 29.335 | (23.990 to 34.680) | <0.001 |
| A | 483 | 35.801 | (30.429 to 41.173) | <0.001 |
| A* | 253 | 42.572 | (37.104 to 48.040) | <0.001 |
| Constant | 3132 | 78.400 | (73.136 to 83.664) | <0.001 |

***Table S4: Grouped WISC score for boys English GCSE attainment***

| Grades attained in GCSE English | N | Coef. | 95% Conf. Interval | p value |
| --- | --- | --- | --- | --- |
| U* | 18 | 0 |  |  |
| G | 23 | -5.437 | (-13.890 to 3.015) | 0.207 |
| F | 84 | -4.016 | (-10.992 to 2.960) | 0.259 |
| E | 239 | -2.322 | (-8.887 to 4.242) | 0.488 |
| D | 532 | 4.148 | (-2.289 to 10.585) | 0.206 |
| C | 899 | 11.496 | (5.102 to 17.889) | <0.001 |
| B | 729 | 19.361 | (12.953 to 25.770) | <0.001 |
| A | 381 | 24.937 | (18.459 to 31.416) | <0.001 |
| A* | 92 | 31.824 | (24.901 to 38.746) | <0.001 |
| Constant | 2997 | 91.611 | (85.280 to 97.942) | <0.001 |

***Table S5: Grouped WISC score for girls English GCSE attainment***

| Grades attained in GCSE English | N | Coef. | 95% Conf. Interval | p value |
| --- | --- | --- | --- | --- |
| U* | 8 | 0 |  |  |
| G | 12 | -7.667 | (-18.774 to 3.441) | 0.176 |
| F | 33 | -8.394 | (-17.984 to 1.196) | 0.086 |
| E | 105 | -4.619 | (-13.544 to 4.306) | 0.310 |
| D | 376 | 2.649 | (-6.046 to 11.344) | 0.550 |
| C | 820 | 9.837 | (1.191 to 18.482) | 0.026 |
| B | 946 | 18.464 | (9.824 to 27.104) | <0.001 |
| A | 660 | 25.677 | (17.022 to 34.333) | <0.001 |
| A* | 210 | 32.129 | (23.363 to 40.895) | <0.001 |
| Constant | 3170 | 88.000 | (79.396 to 96.604) | <0.001 |

***Table S6: Grouped WISC score for boys grouped A-level attainment***

| Grades attained in A-levels | N | Coef. | 95% Conf. Interval | p value |
| --- | --- | --- | --- | --- |
| None* | 602 | 0 |  |  |
| <CCC | 626 | 6.342 | (4.775 to 7.909) | <0.001 |
| <BBB | 324 | 11.111 | (9.220 to 13.002) | <0.001 |
| <AAA | 339 | 14.807 | (12.943 to 16.671) | <0.001 |
| AAA only | 93 | 17.599 | (14.541 to 20.657) | <0.001 |
| AAA+ | 30 | 22.973 | (17.839 to 28.108) | <0.001 |
| AAAA+ | 60 | 24.407 | (20.691 to 28.122) | <0.001 |
| Constant | 2074 | 100.96 | (99.841 to 102.079) | <0.001 |

***Table S7: Grouped WISC score for girls grouped A-level attainment***

| Grades attained in A-levels | N | Coef. | 95% Conf. Interval | p value |
| --- | --- | --- | --- | --- |
| None* | 698 | 0 |  |  |
| <CCC | 620 | 4.612 | (3.205 to 6.020) | <0.001 |
| <BBB | 423 | 9.765 | (8.194 to 11.337) | <0.001 |
| <AAA | 504 | 13.391 | (11.900 to 14.882) | <0.001 |
| AAA only | 152 | 19.448 | (17.166 to 21.731) | <0.001 |
| AAA+ | 19 | 22.238 | (16.308 to 28.168) | <0.001 |
| AAAA+ | 42 | 25.992 | (21.941 to 30.044) | <0.001 |
| Constant | 2458 | 99.341 | (98.376 to 100.306) | <0.001 |

***Instrumental variable (IV) analyses utilising IQ measured at age four***

The following set of analyses utilise a test of cognitive ability that was carried out on a subset of ALSPAC participants at 49 months of age as part of the “Children In Focus” (CIF) clinic subsample. The aims of the CIF was to examine children in more detail than was possible using only questionnaires. Participants were chosen at random from the final six months of ALSPAC births (06/06/92 – 11/12/92) and invited to attend regular clinics, resulting in a sample of 1032 for the 49 month visit at which cognitive ability was measured. Cognitive ability was assessed using the Wechsler Pre-school and Primary Scale of Intelligence – Revised Edition (WPPSI). All scales were administered except for the two optional tests. For more detailed information on the CIF sample and the administering of the WPPSI see the “Children In Focus” data documentation section of the ALSPAC data dictionary available from <http://www.bristol.ac.uk/alspac/external/documents/ALSPAC-documentation-21Mar2015.zip>. The WPPSI full test reliability is high at an average of .96 (see Kamphaus (2005) for detailed information on the WPPSI).

From this sample of 1032 participants who attended the 49 month CIF, WPPSI data is available for 1013. However, further restrictions to those cases that had full information on all covariates as part of this study reduced our analytical sample size to 463. Given the small sample size and the range of SEP measures that we utilise as part of our study (resulting in a total of 320 possible cells) each of our IV analyses is run on each measure of SEP independently. While this does not allow us to consider all measures of SEP together in the same model, these analysis are nevertheless appropriate as they are intended only to test for measurement error within the WISC score. Using the WPPSI as an instrument for the WISC eliminates the WISC of measurement error, on the assumption that measurement error from each test is independent of each other. We present coefficients, 95% confidence intervals, and p values from two-stage least squares instrumental variables regression. For the ease of interpretation we use reversed scales of maternal education and income in this analysis so that each group increase from the baseline unit on all SEP measures represents a lower socioeconomic position than the previous unit. Outcome measures are GCSE grades and A-level groups on an increasing scale so that higher grades represent higher outcome scores.

The unadjusted and instrumented results for each independent measure of SEP in our IV analysis are presented in table S8. Instrumenting for cognitive ability at 49 months attenuates associations between age eight cognitive ability and educational outcomes for each measure of SEP, but every measure still robustly predicts attainment. Maternal education remains the strongest predictor of attainment, and household deprivation the weakest. In terms of attainment, SEP predicts A-levels better than GCSE English and GCSE Math, which are predicted broadly similarly well. Our IV results provide statistical evidence that socioeconomic inequalities in GCSE and A-level attainment persist even when accounting for measurement error in the WISC test. It is important to reiterate that these results are from on a sample of only 463 participants and that such robust findings on a small sample provide weight to our main analysis.

***Table S8: Instrumental variable analysis of family SEP on child educational attainment***

|  | GCSE Maths | | | | GCSE English | | | | A-level | | | |
| --- | --- | --- | --- | --- | --- | --- | --- | --- | --- | --- | --- | --- |
|  | Unadjusted | | Instrumented | | Unadjusted | | Instrumented | | Unadjusted | | Instrumented | |
| Household deprivation | Coef. (95% CI) | p | Coef. (95% CI) | p | Coef. (95% CI) | p | Coef. (95% CI) | p | Coef. (95% CI) | p | Coef. (95% CI) | p |
| 2 | -0.03 (-0.33 to 0.27) | 0.844 | -0.06 (-0.36 to 0.25) | 0.718 | 0.02 (-0.24 to 0.28) | 0.882 | -0.01 (-0.28 to 0.25) | 0.914 | -0.07 (-1.04 to 0.90) | 0.887 | -0.15 (-1.14 to 0.83) | 0.761 |
| 3 | -0.27 (-0.60 to 0.06) | 0.113 | -0.24 (-0.57 to 0.10) | 0.169 | -0.44 (-0.73 to -0.15) | 0.003 | -0.40 (-0.70 to -0.10) | 0.009 | -1.59 (-2.67 to -0.52) | 0.004 | -1.49 (-2.58 to -0.40) | 0.007 |
| 4 | -0.25 (-0.68 to 0.19) | 0.262 | -0.20 (-0.64 to 0.24) | 0.364 | -0.16 (-0.53 to 0.22) | 0.418 | -0.10 (-0.49 to 0.29) | 0.625 | -0.52 (-1.92 to 0.88) | 0.469 | -0.38 (-1.80 to 1.05) | 0.602 |
| 5 - most deprived | -0.48 (-0.89 to -0.07) | 0.020 | -0.44 (-0.85 to -0.02) | 0.038 | -0.47 (-0.82 to -0.12) | 0.009 | -0.41 (-0.78 to -0.05) | 0.027 | -0.80 (-2.12 to 0.52) | 0.234 | -0.66 (-2.00 to 0.68) | 0.332 |
|  |  |  |  |  |  |  |  |  |  |  |  |  |
| Maternal Education |  |  |  |  |  |  |  |  |  |  |  |  |
| A-level | -0.27 (-0.65 to 0.11) | 0.158 | -0.18 (-0.58 to 0.21) | 0.354 | -0.44 (-0.77 to -0.10) | 0.010 | -0.30 (-0.65 to 0.05) | 0.092 | -2.69 (-3.88 to -1.50) | <0.001 | -2.52 (-3.74 to -1.30) | <0.001 |
| O-level | -0.58 (-0.95 to -0.21) | 0.002 | -0.46 (-0.85 to -0.06) | 0.022 | -0.56 (-0.89 to -0.23) | 0.001 | -0.36 (-0.72 to -0.01) | 0.043 | -4.09 (-5.25 to -2.93) | <0.001 | -3.85 (-5.08 to -2.62) | <0.001 |
| CSE/vocational | -1.08 (-1.50 to -0.66) | <0.001 | -0.90 (-1.36 to -0.43) | <0.001 | -0.96 (-1.33 to -0.58) | <0.001 | -0.67 (-1.09 to -0.26) | 0.002 | -5.01 (-6.34 to -3.68) | <0.001 | -4.66 (-6.11 to -3.22) | <0.001 |
|  |  |  |  |  |  |  |  |  |  |  |  |  |
| Social class |  |  |  |  |  |  |  |  |  |  |  |  |
| III-NM | -0.28 (-0.59 to 0.02) | 0.072 | -0.20 (-0.52 to 0.11) | 0.203 | -0.27 (-0.54 to -0.01) | 0.044 | -0.17 (-0.45 to 0.10) | 0.222 | -1.87 (-2.84 to -0.90) | <0.001 | -1.66 (-2.65 to -0.66) | 0.001 |
| III-M | -0.40 (-0.73 to -0.07) | 0.018 | -0.27 (-0.61 to 0.08) | 0.129 | -0.44 (-0.72 to -0.15) | 0.003 | -0.27 (-0.58 to 0.04) | 0.085 | -2.44 (-3.49 to -1.40) | <0.001 | -2.08 (-3.18 to -0.98) | <0.001 |
| IV & V | -0.66 (-1.05 to -0.26) | 0.001 | -0.46 (-0.88 to -0.03) | 0.035 | -0.74 (-1.08 to -0.39) | <0.001 | -0.48 (-0.85 to -0.10) | 0.013 | -3.17 (-4.43 to -1.90) | <0.001 | -2.60 (-3.95 to -1.25) | <0.001 |
|  |  |  |  |  |  |  |  |  |  |  |  |  |
| Family income at 7 |  |  |  |  |  |  |  |  |  |  |  |  |
| £300-399 | -0.30 (-0.59 to -0.02) | 0.039 | -0.24 (-0.53 to 0.05) | 0.109 | -0.04 (-0.29 to 0.21) | 0.732 | 0.04 (-0.22 to 0.30) | 0.749 | -0.34 (-1.26 to 0.57) | 0.463 | -0.17 (-1.11 to 0.77) | 0.719 |
| £200-299 | -0.52 (-0.84 to -0.19) | 0.002 | -0.44 (-0.78 to -0.10) | 0.011 | -0.41 (-0.69 to -0.12) | 0.005 | -0.30 (-0.60 to 0.00) | 0.052 | -2.17 (-3.22 to -1.11) | <0.001 | -1.95 (-3.03 to -0.86) | <0.001 |
| < £199 | -0.70 (-1.09 to -0.30) | 0.001 | -0.55 (-0.97 to -0.13) | 0.010 | -0.70 (-1.05 to -0.35) | <0.001 | -0.50 (-0.87 to -0.12) | 0.009 | -2.26 (-3.55 to -0.98) | 0.001 | -1.86 (-3.20 to -0.51) | 0.007 |

Coef, regression coefficient; 95% CI, 95% confidence interval; p, p value; CSE, certificate of secondary education; III-NM, Social class III (non-manual); III-M, Social class III (manual); IV, Social class IV; V, Social class V.

***Errors in variables analysis***

In order to be further secure that measurement error in the WISC does not invalidate our findings we ran an errors in variables regression analysis. This approach allows us to specify varying levels of measurement error and therefore ascertain if the results of our main analysis are biased by WISC measurement error; if measurement error exists within the WISC then our SEP results will be biased given their correlation with the WISC. To conduct this analysis we specify a highly conservative reliability value for the WISC test of 0.7. This is based upon the reliability of the lowest scoring subtest that is used in the ALSPAC age eight WISC test and is conservative because full scale reliability is higher than the lowest individual subtest reliability and becomes more reliable the more scales that are used due to the way that full scale reliability scores are computed (Sattler, 2009). Because we are not restricted to a smaller sample as with the IV analysis, these models utilise our full sample and are presented as multivariate regression analyses for each attainment variable alike to our main regression analysis in Table 4. The results, presented in table S9, provide further evidence that our main results are robust to measurement error of 30% in the WISC; a highly conservative estimate given the test reliability. Compared to our main regression results associations between SEP measures and educational outcome are generally attenuated but remain. Maternal education remains the most strongly associated SEP measure with attainment, and as with the IV results in table S8 household deprivation suffers the least attenuation when accounting for measurement error. Similarly to the IV regression A-level attainment remains the best predicted educational outcome by family SEP. Our instrumental variables and errors in regression analyses provide further support for our main analysis that attainment diverges based on SEP in ways that cannot be explained by measurement error alone.

***Table S9: Error in variables regression results of social inequalities in educational attainment (WISC reliability=0.7).***

|  | GCSE Maths | | GCSE English | | A-level |  |
| --- | --- | --- | --- | --- | --- | --- |
| Household deprivation | Coef. (95% CI) | p | Coef. (95% CI) | p | Coef. (95% CI) | p |
| 2 | -0.05 (-0.13 to 0.04) | 0.251 | -0.10 (-0.18 to -0.02) | 0.020 | -0.30 (-0.60 to 0.00) | 0.048 |
| 3 | -0.08 (-0.18 to 0.01) | 0.092 | -0.20 (-0.29 to -0.11) | <0.001 | -0.77 (-1.11 to -0.44) | <0.001 |
| 4 | -0.21 (-0.32 to -0.10) | <0.001 | -0.11 (-0.22 to 0.00) | 0.052 | -0.76 (-1.16 to -0.36) | <0.001 |
| 5 - most deprived | -0.36 (-0.48 to -0.24) | <0.001 | -0.24 (-0.35 to -0.13) | <0.001 | -0.77 (-1.18 to -0.37) | <0.001 |
|  |  |  |  |  |  |  |
| Maternal Education | |  |  |  |  |  |
| A-level | -0.08 (-0.19 to 0.03) | 0.154 | -0.08 (-0.18 to 0.03) | 0.140 | -1.35 (-1.73 to -0.96) | <0.001 |
| O-level | -0.17 (-0.29 to -0.06) | 0.004 | -0.24 (-0.36 to -0.13) | <0.001 | -2.34 (-2.74 to -1.93) | <0.001 |
| CSE/vocational | -0.30 (-0.44 to -0.17) | <0.001 | -0.36 (-0.49 to -0.23) | <0.001 | -2.54 (-3.01 to -2.06) | <0.001 |
|  |  |  |  |  |  |  |
| Social class | |  |  |  |  |  |
| III-NM | 0.04 (-0.06 to 0.13) | 0.429 | 0.04 (-0.05 to 0.130) | 0.358 | -0.41 (-0.74 to -0.09) | 0.013 |
| III-M | -0.13 (-0.23 to -0.03) | 0.010 | -0.20 (-0.30 to -0.11) | <0.001 | -1.13 (-1.48 to -0.79) | <0.001 |
| IV & V | -0.19 (-0.31 to -0.08) | 0.001 | -0.22 (-0.33 to -0.11) | <0.001 | -0.93 (-1.33 to -0.52) | <0.001 |
|  |  |  |  |  |  |  |
| Family income at 7 | |  |  |  |  |  |
| £300-399 | -0.03 (-0.11 to 0.06) | 0.550 | -0.05 (-0.13 to 0.03) | 0.237 | -0.13 (-0.43 to 0.17) | 0.400 |
| £200-299 | -0.14 (-0.24 to -0.05) | 0.003 | -0.12 (-0.21 to -0.03) | 0.008 | -0.66 (-0.99 to -0.32) | <0.001 |
| < £199 | -0.24 (-0.36 to -0.13) | <0.001 | -0.26 (-0.37 to -0.15) | <0.001 | -0.91 (-1.32 to -0.51) | <0.001 |
|  |  |  |  |  |  |  |
| WISC | 0.08 (0.08 to 0.08) | <0.001 | 0.06 (0.05 to 0.06) | <0.001 | 0.17 (0.16 to 0.18) | <0.001 |

Coef, regression coefficient; 95% CI, 95% confidence interval; p, p value; CSE, certificate of secondary education; III-NM, Social class III (non-manual); III-M, Social class III (manual); IV, Social class IV; V, Social class V; WISC, WISC cognitive ability score.

Note that for tables S10 to S15 certain GCSE and A-level grades were grouped together to overcome small sample issues caused by the low number of cases with maternal WISC score available. These analyses are based upon a reduced sample of 1676 cases due to missing data on maternal cognitive ability. Maternal cognitive ability was assessed at the age 15 child clinic using the Wechsler Abbreviated Scale of Intelligence (WASI) test, a short form cognitive test designed for adults (Wechsler, 1999).

***Table S10: Grouped maternal WISC score for Maths GCSE attainment***

| Grade attained in GCSE Maths | N | Coef. | 95% Conf. Interval | p value |
| --- | --- | --- | --- | --- |
| U/G/F* | 97 | 0 |  |  |
| E/D | 406 | 3.887 | (1.024 to 6.749) | 0.008 |
| C | 663 | 7.202 | (4.448 to 9.955) | <0.001 |
| B | 523 | 11.651 | (8.851 to 14.452) | <0.001 |
| A/A* | 615 | 14.881 | (12.113 to 17.648) | <0.001 |
| Constant | 2304 | 89.165 | (86.593 to 91.737) | <0.001 |

***Table S11: Grouped maternal WISC score for Maths GCSE attainment accounting for child WISC score***

|  | N | Coef. | 95% Conf. Interval | p value |
| --- | --- | --- | --- | --- |
| Child WISC | 2304 | 0.179 | (0.139 to 0.220) | <0.001 |
| Grade attained in GCSE Maths |  |  |  |  |
| U/G/F* | 97 | 0 |  |  |
| E/D | 406 | 1.937 | (-0.915 to 4.789) | 0.183 |
| C | 663 | 3.694 | (0.869 to 6.518) | 0.010 |
| B | 523 | 6.906 | (3.948 to 9.865) | <0.001 |
| A/A* | 615 | 8.631 | (5.560 to 11.701) | <0.001 |
| Constant | 2304 | 74.331 | (70.122 to 78.540) | <0.001 |

***Table S12: Grouped maternal WISC score for English GCSE attainment***

| Grade attained in GCSE English | N | Coef. | 95% Conf. Interval | p value |
| --- | --- | --- | --- | --- |
|  |  |  |  |  |
| U/G/F* | 43 | 0 |  |  |
| E/D | 389 | 6.201 | (2.139 to 10.262) | 0.003 |
| C | 657 | 8.508 | (4.530 to 12.487) | <0.001 |
| B | 677 | 13.569 | (9.594 to 17.543) | <0.001 |
| A/A* | 595 | 17.987 | (13.996 to 21.977) | <0.001 |
| Constant | 2361 | 87.000 | (83.146 to 90.854) | <0.001 |

***Table S13: Grouped maternal WISC score for English GCSE attainment accounting for child WISC score***

|  | N | Coef. | 95% Conf. Interval | p value |
| --- | --- | --- | --- | --- |
| Child WISC | 2361 | 0.188 | (0.150 to 0.225) | <0.001 |
| Grade attained in GCSE English | | | |  |
| U/G/F* | 43 | 0 |  |  |
| E/D | 389 | 4.817 | (0.824 to 8.810) | 0.018 |
| C | 657 | 5.524 | (1.576 to 9.472) | 0.006 |
| B | 677 | 9.100 | (5.099 to 13.100) | <0.001 |
| A/A* | 595 | 12.052 | (7.959 to 16.145) | <0.001 |
| Constant | 2361 | 70.927 | (65.946 to 75.907) | <0.001 |

***Table S14: Grouped maternal WISC score for A-level attainment***

| Grades attained in A-levels | N | Coef. | 95% Conf. Interval | p value |
| --- | --- | --- | --- | --- |
| None* | 479 | 0 |  |  |
| <CCC | 509 | 2.309 | (0.719 to 3.898) | 0.004 |
| <BBB | 312 | 5.368 | (3.552 to 7.185) | <0.001 |
| <AAA | 387 | 8.287 | (6.581 to 9.994) | <0.001 |
| AAA only | 111 | 11.575 | (8.945 to 14.205) | <0.001 |
| AAA+ | 67 | 10.797 | (7.540 to 14.053) | <0.001 |
| Constant | 1865 | 96.263 | (95.122 to 97.404) | <0.001 |

***Table S15: Grouped maternal WISC score for A-level attainment accounting for child WISC score***

|  | N | Coef. | 95% Conf. Interval | p value |
| --- | --- | --- | --- | --- |
| Child WISC | 1865 | 0.176 | (0.134 to 0.218) | <0.001 |
| Grades attained in A-levels | | |  |  |
| None* | 479 | 0 |  |  |
| <CCC | 509 | 1.506 | (-0.067 to 3.079) | 0.061 |
| <BBB | 312 | 3.500 | (1.660 to 5.339) | <0.001 |
| <AAA | 387 | 5.902 | (4.132 to 7.673) | <0.001 |
| AAA only | 111 | 8.321 | (5.623 to 11.019) | <0.001 |
| AAA+ | 67 | 6.314 | (2.940 to 9.688) | <0.001 |
| Constant | 1865 | 78.409 | (74.005 to 82.812) | <0.001 |

***Table S16: School differences in low/high ability attainment***

|  | State | | | | Fee paying | | | |
| --- | --- | --- | --- | --- | --- | --- | --- | --- |
|  | Q1 - lowest | Q2 | Q3 | Q4 - highest | Q1 - lowest | Q2 | Q3 | Q4 - highest |
| None | 375 (54.19) | 383 (37.7) | 296 (25.74) | 151 (13.73) | 10 (29.41) | 8 (11.76) | 10 (10.75) | 3.35 (3.35) |
| <CCC | 202 (29.19) | 310 (30.51) | 356 (30.96) | 250 (22.73) | 11 (32.35) | 19 (27.94) | 15 (16.13) | 6.7 (10.06) |
| <BBB | 67 (9.68) | 157 (15.45) | 220 (19.13) | 222 (20.18) | 9 (26.47) | 3 (4.41) | 14 (15.05) | 14.53 (24.58) |
| <AAA | 40 (5.78) | 137 (13.48) | 211 (18.35) | 296 (26.91) | 3 (8.82) | 26 (38.24) | 38 (40.86) | 36.87 (61.45) |
| AAA only | 7 (1.01) | 22 (2.17) | 47 (4.09) | 93 (8.45) | 1 (2.94) | 11 (16.18) | 9 (9.68) | 25.14 (86.59) |
| AAA+ | 1 (0.14) | 4 (0.39) | 7 (0.61) | 31 (2.82) | 0 (0.00) | 0 (0.00) | 1 (1.08) | 2.23 (88.83) |
| AAAA+ | 0 (0.00) | 3 (0.30) | 13 (1.13) | 57 (5.18) | 0 (0.00) | 1 (1.47) | 6 (6.45) | 11.17 (100) |

***Table S17: Regression analysis of school type and WISC prediction of A-level attainment***

| Covariate | N | Coef. | 95% Conf. Interval | P>t |
| --- | --- | --- | --- | --- |
|  |  |  |  |  |
| School type | |  |  |  |
| State* | 2932 | 0 |  |  |
| Fee paying | 166 | 0.759 | (0.558 to 0.960) | <0.001 |
| WISC @ 8 score | |  |  |  |
| per unit | 3098 | 0.04 | (0.037 to 0.043) | <0.001 |
| Constant | 3098 | -2.835 | (-3.169 to -2.501) | <0.001 |

***Table S18: Odds Ratios of attaining 5+ A*-C GCSEs including English and Maths by family socioeconomic position***

| Covariate |  | OR (95% CI) | p |
| --- | --- | --- | --- |
| Household deprivation score | 1 - Least deprived | |  |
|  | 2 | 0.89 (0.72 to 1.11) | 0.308 |
|  | 3 | 0.73 (0.58 to 0.93) | 0.009 |
|  | 4 | 0.8 (0.6 to 1.05) | 0.106 |
|  | 5 - Most deprived | 0.54 (0.41 to 0.71) | <0.001 |
|  |  |  |  |
| Maternal education | CSE/Vocational | |  |
|  | O level | 0.53 (0.37 to 0.77) | 0.001 |
|  | A level | 0.33 (0.23 to 0.47) | <0.001 |
|  | Degree | 0.25 (0.17 to 0.37) | <0.001 |
|  |  |  |  |
| Social class | I & II |  |  |
|  | III-NM | 1.07 (0.84 to 1.38) | 0.578 |
|  | III-M | 0.62 (0.49 to 0.79) | <0.001 |
|  | IV & V | 0.56 (0.42 to 0.73) | <0.001 |
|  |  |  |  |
| Family income at 7 | < £199 |  |  |
|  | £200-£299 | 0.96 (0.78 to 1.18) | 0.687 |
|  | £300-£399 | 0.78 (0.62 to 0.97) | 0.026 |
|  | £400+ | 0.57 (0.44 to 0.75) | <0.001 |
|  |  |  |  |
| WISC | | 1.08 (1.07 to 1.09) | <0.001 |

OR, Odds Ratio; 95% CI, 95% confidence interval; p, p value; CSE, certificate of secondary education; III-NM, Social class III (non-manual); III-M, Social class III (manual); IV, Social class IV; V, Social class V; WISC, WISC cognitive ability score.

***Regression towards the mean (RTM) in ALSPAC cognitive tests***

Regression towards the mean (RTM) is a statistical phenomenon that can explain some of the narrowing of *trajectories* between groups of high and low ability children from differing SEP backgrounds that has been observed in previous studies. Jerrim & Vignoles (2013) provide a detailed explanation of RTM and present an RTM adjusted analysis to demonstrate that the narrowing of cognitive ability trajectories between high ability low SEP and low ability high SEP children is not as extreme as has previously been suggested (Feinstein, 2003). However, as we do not model *trajectories* of cognitive ability but associations between cognitive ability at age eight and educational attainment at ages 16 and 18 RTM it is the issue of measurement error within the WISC (which is a distinct statistical issue to RTM) that threatens the validity of our main findings rather than RTM itself. This is because measurement error is likely to be greater in the tails of the ability distribution, particularly for high SEP/low ability and low SEP/high ability children as these values are the furthest from their SEP group means. As such, it is possible that our results in Tables 2 and 3 may suffer some bias as they focus on children within the top 10% of the ability distribution. Given that our main results in Table 4 include all ALSPAC children available in our analytical sample and not just those at the tail of the ability distribution there is less concern of such measurement error bias; indeed our results appear robust to this given the sensitivity analysis in tables S8 and S9.

Nevertheless, an examination of RTM within ALSPAC cognitive ability trajectories is of interest. Cognitive ability at age 15 is available on a relatively large number of ALSPAC children in our analytical sample (n=2632). However, age 15 ability is measured using a different test (Wechsler Abbreviated Scale of Intelligence; WASI) to the age eight ability (WISC) and the use of different tests is cautioned against (see Axelrod, 2002; Strauss et al, 2006). This caution is compounded in ALSPAC due to scoring errors with the age 15 data whereby mean cognitive ability is significantly lower at age 15 than age eight (13.5 points in our analytical sample) rendering comparisons potentially invalid. In order to attempt an exploratory analysis of the extent of RTM between ages eight and 15, results from both tests were standardised to z scores with a mean of zero and standard deviation of one. Figure S2 shows the regression to the mean of standardised ALSPAC cognitive test scores at age eight (WISC) and age 15 (WASI). Figure S2 presents the change in z score between ages eight and 15 that is attributable to RTM for children in the first and tenth deciles of cognitive ability at age eight, separated by SEP background (where low SEP is defined as child’s mother holding CSE/vocational qualifications, and high SEP defined as university degree). The most extreme deciles were selected on the basis that our initial analysis in Tables 2 and 3 focussed on children in the top 10% of the WISC distribution. We utilise maternal education as the measure of SEP because it has the largest association to cognitive ability of our SEP measures and can therefore be expected to suffer a greater proportion of RTM than these. There is indeed evidence for RTM between age eight and 15 cognitive scores in ALSPAC - proportionately more so for the low SEP/high ability and high SEP/low ability groups - but differences between the groups remain. As such, any analysis of cognitive ability *trajectories* within ALSPAC should be mindful of RTM in addition to concerns of the scoring issues inherent in the age 15 WASI data and the interchangeable use of the age eight WISC and the age 15 WASI.

***Figure S2: Plot of the expected pre- and post-test means (z scores)***

***References***

Axelrod BN. (2002). Validity of the Wechsler abbreviated scale of intelligence and other very short forms of estimating intellectual functioning. Assessment, Mar;9(1):17-23.

Kamphaus RW. (2005). Clinical Assessment of Child and Adolescent Intelligence. Springer: New York.

Satller JM, Ryan JJ (Eds.). (2009). Assessment with the WAIS-IV. Sattler publisher: California.

Strauss E, Sherman EMS, Spreen O. (2006). A Compendium of Neuropsychological Tests: Administration, Norms, and Commentary. Oxford University Press.

Wechsler, D. (1999). Wechsler Abbreviated Scale of Intelligence. The Psychological Corporation: Harcourt Brace & Company. New York, NY.
